# Biodistribution of Idursulfase Formulated for Intrathecal Use (Idursulfase-IT) in Cynomolgus Monkeys after Intrathecal Lumbar Administration

**DOI:** 10.1371/journal.pone.0164765

**Published:** 2016-10-20

**Authors:** Jou-Ku Chung, Eilish Brown, Bob Crooker, Kathleen J. Palmieri, Thomas G. McCauley

**Affiliations:** Shire, Lexington, Massachusetts, United States of America; Hungarian Academy of Sciences, HUNGARY

## Abstract

Enzyme replacement therapy with intravenous idursulfase (recombinant iduronate-2-sulfatase) is approved for the treatment of Hunter syndrome. Intravenous administration does not, however, treat the neurological manifestations, due to its low central nervous system bioavailability. Using intrathecal-lumbar administration, iduronate-2-sulfatase is delivered directly to the central nervous system. This study investigates the central nervous system biodistribution of intrathecal-lumbar administered iduronate-2-sulfatase in cynomolgus monkeys. Twelve monkeys were administered iduronate-2-sulfatase in one 30 mg intrathecal-lumbar injection. Brain, spinal cord, liver, and kidneys were collected for iduronate-2-sulfatase concentration (measured by an enzyme linked immunosorbent assay) and enzyme activity measurement (via a method utilizing 4-methylumbelliferyl-α-iduronate-2-sulfate) at 1, 2, 5, 12, 24, and 48 hours following administration. The tissue enzyme linked immunosorbent assay confirmed iduronate-2-sulfatase uptake to the brain, spinal cord, kidneys, and liver in a time-dependent manner. In spinal cord and brain, iduronate-2-sulfatase appeared as early as 1 hour following administration, and peak concentrations were observed at ~2 and ~5 hours. Iduronate-2-sulfatase appeared in liver and kidneys 1 hour post intrathecal-lumbar dose with peak concentrations between 5 and 24 hours. Liver iduronate-2-sulfatase concentration was approximately 10-fold higher than kidney. The iduronate-2-sulfatase localization and enzyme activity in the central nervous system, following intrathecal administration, demonstrates that intrathecal-lumbar treatment with iduronate-2-sulfatase may be considered for further investigation as a treatment for Hunter syndrome patients with neurocognitive impairment.

## Introduction

Hunter syndrome is an X-linked disease caused by a deficiency or absence of the enzyme iduronate-2-sulfatase (I2S), a lysosomal storage enzyme required for the degradation of glycosaminoglycans [1]. It has an incidence of about 1 in 170,000 male births [2]. The enzyme deficiency causes the glycosaminoglycans, heparan sulfate and dermatan sulfate, to accumulate in the lysosomes, contributing to the signs and symptoms of the disease. About two-thirds of patients with Hunter syndrome will develop neurologic involvement with associated cognitive decline [3, 4]. There is currently no cure for this disease, however, intravenous enzyme replacement therapy with recombinant human I2S (idursulfase, Elaprase^®^ Shire, Lexington, MA) is an approved treatment in over 50 countries for Hunter syndrome. Idursulfase treatment significantly improves some somatic manifestations of the disease [5, 6], but does not penetrate the blood–brain barrier at doses sufficient to affect the cognitive decline observed in severe disease [6, 7]. It is therefore important to find an effective method of drug delivery for therapy to the brain.

When an idursulfase formulation suitable for intrathecal-lumbar (IT-L) (idursulfase-IT) infusion was administered monthly to cynomolgus monkeys (in addition to weekly intravenous idursulfase infusions) over a 6-month period, exogenous I2S was observed to be distributed throughout the brain and spinal cord, and it was generally well tolerated.[8]. A later study also demonstrated that intracerebroventricular and IT-L administration of idursulfase-IT to dogs and cynomolgus monkeys resulted in the enzyme being detected widely throughout the brain [9].

Xie et al, using 1-, 10-, and 30-mg doses of idursulfase-IT in cynomolgus monkeys demonstrated that high maximum concentrations of I2S were immediately observed in the cerebrospinal fluid (CSF), with an elimination half-life between 5.9–10 hours, in contrast to intravenously administered idursulfase [10]. The CSF pharmacokinetic profiles at different I2S doses were similar and the dose/exposure relationship was proportional. Calias et al [9], using an *I2S* gene knockout mouse model of Hunter syndrome observed that compared with untreated mice, intrathecally administered idursulfase treatment decreased cellular vacuolation in parts of the central nervous system such as, the surface cerebral cortex, cerebellum, and thalamus, indicating that I2S was active within the neural tissue.

Recently an idursulfase-IT formulated for human intrathecal administration was investigated in a phase I/II trial in patients with Hunter syndrome who were also receiving IV idursulfase. After 6 months, mean CSF GAG concentrations were reduced by approximately 90% in the 10-mg and 30-mg dose groups and approximately 80% in the 1-mg group [11].

The objective of this study was to investigate the dynamics of I2S distribution to the spinal cord and the cerebral compartments; and to investigate its uptake from the CNS to the general circulation and peripheral organs after a single IT-L administration of idursulfase-IT in cynomolgus monkeys.

The cynomolgus monkey was chosen as the test system for this study because of its established role as a model for histological, toxicological, and pharmacological studies in a large animal species. In addition they have been used in previous studies of idursulfase-IT in the CNS [8–10]. IT-L administration has a number of advantages over intracerebroventricular administration as it is less invasive, is a routine clinical procedure, and additionally spinal administration may help reduce the levels of GAGs in the spinal cord itself.

## Materials and Methods

### Ethics Statement

Care of the animals was conducted in accordance with the guidelines, *Guide for the Care and Use of Laboratory Animals*, *United States Department of Health and Human Services*, *No 86–23*, and the *Animal Welfare Act (9 CFR Part 3); USDA No*. *34-R-0025)*. The studies were performed at Northern Biomedical Research, Inc. (NBR; Muskegon, MI), an organization accredited by the Association for Assessment and Accreditation of Laboratory Animal Care. The studies were approved by the Institutional Animal Care and Use Committee of NBR (cynomolgus monkey study protocol number 047–028).

### Experimental Animals

Cynomolgus monkeys (*Macaca fasicularis*) (Covance Research Products, Emeryville, CA) were singly housed in a colony room under a 12-hour light-dark cycle, with 50% (± 30%) humidity, at 22°C (± 2°C). The cages were sized 25–27 x 24 x 36 inches, with 4.2 square feet of floor space, and were >12 cubic feet in volume. Appropriate food, water, treats, and vitamin supplements were provided, and animals were given access to environmental enrichment such as approved toys, swings, perches, mirrors, television, or music to promote psychological well-being. Every effort was made to minimize pain, discomfort, and suffering through the use of appropriate methods and agents for analgesia, anesthesia, and euthanasia. All animals were under the care and supervision of a veterinarian.

The animals were individually housed in stainless steel cages in two rooms at NBR. Temperature and humidity were recorded daily. The temperature and relative humidity values of the animal rooms were 20°–23°C (68°–73°F) and 41%–78% for one room, and 19°–24°C (66–75°F) and 27%–69% for the other. Each room was equipped with an automatic timer and the animals received 12 hours of light and darkness each day. The light-dark cycle was interrupted for additional observations or procedures; these interruptions were recorded in the raw data. Room airflow was set to provide at least 10 air changes per hour. Excrement pans were cleaned and fresh wood chips were added daily. The animal cages were washed and sanitized every two weeks. The animals received environmental enrichment per the current NBR Program of Animal Care.

Twenty-five biscuits of PMI Certified Primate Diet 5048 (PMI Nutrition, Arden Hills, MN) were placed in the animals’ feeders each day. The manufacturer analyzed the feed and no contaminants were shown to exist in the food at levels that would be expected to interfere with the integrity of this study. A copy of the feed analysis for each feed lot used in the study is kept in the study records. The animals’ diet was supplemented with vitamin C (manufacturer and lot numbers were recorded in the raw data) on a weekly basis.

Food consumption data was collected daily, beginning prior to surgery and continuing throughout the dosing period. Food was withheld at least 12 hours prior to general anesthesia (e.g., surgery). With the approval of the study director, food was not withheld 12 hours prior to repair surgeries. The water utilized in this study (Muskegon municipal water) was provided via a filtered automatic water system *ad libitum*. The water was analyzed annually for heavy metals, trihalomethanes, PCBs, and pesticides, and analyzed quarterly for microbiological contaminants. No contaminants were shown in the water at concentrations expected to interfere with the purpose of this study.

Animals were observed at least twice daily for morbidity and mortality beginning on the first day of dosing. No idursulfase-related morbidity or mortality was observed during the study. Clinical signs were recorded at least twice daily post surgery throughout the study. The animals were observed for signs of clinical effects, illness, and/or death. Clinical signs unrelated to idursulfase included wounds (i.e. wound on back of neck from collar, wound on back of head), incision/delivery system observations (i.e. seroma over cisterna magna port, reddened area over lumbar port), paresis, motor deficit, loose stools, and emesis. Incision/delivery system observations were noted for various animals. The majority of the observations were noted in the week prior to the dosing period. Clinical observations noted included paresis (Days -7 to -4), motor deficit (Days -3 to 3, 6, and 7), incision/delivery system observations (Day -2), few feces (Day -5), and wounds (Days 4 to 6) occurring in one animal each.

### Study Design

Six groups of 2 animals (1 male and 1 female/group; median weight 2.85 kg, range 2.70–3.14 kg) were each administered 30 mg of idursulfase-IT in a single IT-L injection. The animals had been dosed previously with idursulfase (0.5 mg/kg, IV or 1, 10 and 30 mg IT-L) in the pharmacokinetic arm of the study, which has been previously published [10]. One additional control group consisting of 2 animals were untreated. There was a 7-day washout of dosed I2S after the pharmacokinetic study, and all reused animals had a patent catheter system in place.

### Surgery and Surgical Recovery

At catheter implantation, the animals were pretreated with a subcutaneous injection of atropine sulfate at a dose of 0.04 mg/kg. Approximately 15 minutes later, an intramuscular (IM) dose of 8 mg/kg of ketamine HCl was provided to induce sedation. The animals were masked to a surgical plane of anesthesia, intubated, and maintained on approximately 1 L/min of oxygen and 2% isoflurane. The anesthetic gases and mixtures were varied as required by each individual animal. Prednisolone sodium succinate IV, 30 mg/kg, and flunixin meglumine IM, 2 mg/kg, were administered prior to surgery.

For IT-L catheter implantation, an incision was made over the dorsal process of the lumbar spine at L_4_ or L_5_. The muscle was dissected and a hemilaminectomy was made for the insertion of a tapered polyurethane catheter (0.9 mm OD and 0.5 mm ID tapered/sheathed open end catheter with side holes, P/N 69–2320). The catheter was advanced to the area of the thoraco-lumbar junction. The proximal end of the IT-L catheter was attached to a subcutaneous access port (P.A.S. PORT^®^ Elite plastic/titanium portal with ULTRA-LOCK^®^ connector, P/N 69–2316). Proper catheter placement was confirmed with the aid of a myelogram with Isovue 300 (0.8 ml, Bracco Diagnostics, Inc, Princeton, NJ). The tissue layers were closed with sutures and tissue adhesive was applied.

Upon recovery from anesthesia, the animals were provided butorphanol tartrate IM, 0.05 mg/kg, for analgesia and placed on post-surgical antibiotic ceftiofur sodium IM, 5.0 mg/kg, bid (one injection prior to surgery followed by three injections). Idursulfase-IT dosing started at least seven days after surgery.

### Administration of I2S

Recombinant human I2S was expressed and purified from a human-derived cell line [6] and was provided in IT formulation (idursulfase-IT) [8]. This was diluted with 154 mM NaCl, 0.005% polysorbate 20, pH 6.0 to bring to a concentration of 30 mg/mL and stored at -60°C. The formulation was warmed to room temperature on the bench top before using.

**Fig 1:**
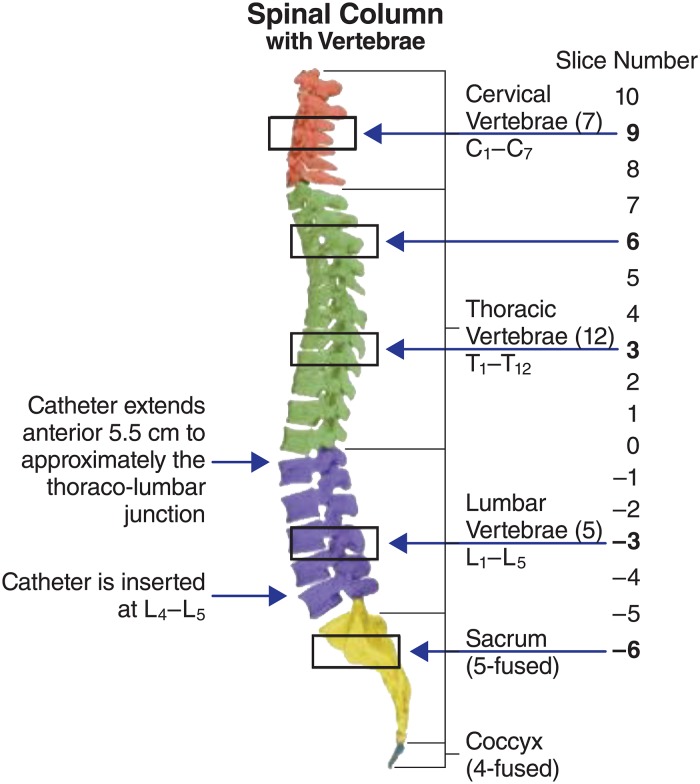
Localization of catheter opening and spinal cord slice relation to the vertebrae of the spine. The tip of the catheter was located at ~T_12_ or L_1_. In the procedure of sectioning, a slice at the tip of the catheter was labeled as 0. From this point, the spinal cord was cut in 1 cm sections in both directions. The slices posterior to 0 were negative and the slices anterior to 0 were positive.

Idursulfase-IT was administered intrathecally through a catheter implanted at the lumbar spine level L_4_ or L_5_ (Fig 1). The IT-L route of administration was selected because this is the anticipated route for human administration. The IT-L dosing was performed by hand and the rate of administration of the idursulfase-IT solution was approximately 0.5 mL/min to give a final dose volume of 1.0 mL per animal (total idursulfase-IT dose of 30 mg). This was followed by 0.5 mL of phosphate buffered saline to flush the dose from the catheter system. The total duration of administration was approximately 2–3 minutes. Each animal received 30 mg of idursulfase-IT.

### Sample Collection and Analysis

One group of two animals (1 male, 1 female) were sacrificed as each time point of 1, 2, 5, 12, 24, and 48 hours after IT-L administration. The brain, spinal cord, liver, kidneys and other organs and tissues were collected for test measurements and immunohistochemical analyses. Blood serum and CSF were collected prior to dosing and at each time point.

### Necropsy and Tissue Collection

All animals, including the controls, were sacrificed. For euthanization, all animals were sedated with ketamine HCl (IM, 8 mg/kg), were maintained on an isoflurane/oxygen mixture, and received an IV bolus of heparin sodium (200 IU/kg). Prior to necropsy, the animals were perfused via the left cardiac ventricle with 0.001% sodium nitrite in saline.

#### Brain

**Fig 2:**
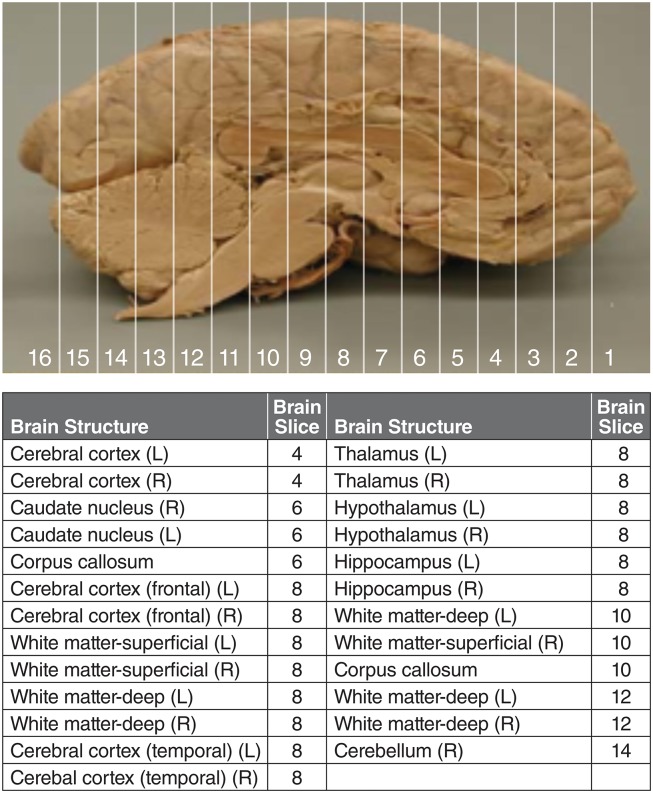
Coronal slicing the monkey brain. The brain was cut into 16 slices in a brain matrix from frontal to distal direction at 3 mm coronal slice thickness. R, right; L, left.

At the time of sacrifice, the brain was cut into 16 slices in a brain matrix at 3 mm coronal slice thickness (Fig 2). The first slice and every other slice thereafter (odd number slices in Fig 2) were fixed in 10% neutral buffered formalin (NBF) for immunohistochemical analysis (not discussed further in this paper). The second slice and every other slice thereafter (the even number slides in Fig 2) were dissected for selected brain tissues or structures. The brain samples were frozen and stored at -60°C or below for I2S concentration analyses. “Superficial” samples were taken from the top 3 mm or less of brain tissue, and “deep” samples were taken from a depth of 3 mm or greater.

#### Spinal Cord

The spinal cord (cervical, thoracic, and lumber) was cut into 1 cm sections (Fig 1). A slice dissected at the tip of the catheter approximately T12 to L1) was designated point 0. From the 0 point, the spinal cord was cut in 1 cm sections in both directions. The slices posterior to 0 are negative (lumbar region) and the slices anterior to 0 are positive (thoracic and cervical). The first slice and every second slice thereafter were fixed in NBF. The second slice and every second slice thereafter were frozen and stored at -60°C or below until analysis for I2S concentrations.

#### Kidney and Liver

The kidney and liver samples were collected, and fixed in 10% neutral buffered formalin and frozen at -60°C or below for analysis. The bioanalytical samples were shipped for analysis on dry ice and the samples for immunohistochemistry were shipped at room temperature to Shire.

### Pre-Dose I2S Level Determination

In order to determine the background, endogenous levels of I2S in these animals, CSF and serum samples were collected prior to idursulfase-IT dosing in all animals (pre-dose or Time 0). Two naïve control female animals were sacrificed and perfused. The brain, spinal cord, and kidneys of these 2 control animals were harvested, sectioned and shipped as described above. Liver pre-dose samples were obtained from three different treatment-naïve monkeys. These three animals had been implanted with catheters which lost patency, so could not be used in the study. As a result, these were used to provide liver biopsies.

### Tissue Sample Analysis

#### Tissue Preparation

Tissue samples were homogenized in lysis buffer formulated with 10mM Tris, 5mM EDTA, 0.1% IGEPAL^®^ (Sigma-Aldrich, St. Louis, MO) and supplemented with Alpha Complete Protease Inhibitor mini-tablets (Roche Diagnostics, Indianapolis, IN). One mL of lysis buffer was added to the tubes per 0.25 g of tissue. Precellys 24 tissue homogenizers (Krackeler Scientific, Albany, NY) with 2.8 mm or 1.4 mm (brain punches) ceramic beads homogenized at 6500 rpm for 40 seconds were used to homogenize the samples. Once homogenized, samples were subjected to 5 freeze-thaw cycles using an ethanol/dry ice bath and a 37°C water bath. Tissue debris was pelleted twice by centrifugation at 4°C for 10 minutes at 14,000 rpm, and supernatants were collected and stored at –80°C until assayed. For brain punches, due to their small tissue sample size, only one centrifugation step was performed.

#### Analysis for I2S Activity by 4-methylumbelliferyl-α-iduronate-2-sulfate (4-MUF) Method

I2S activity in tissue extracts, CSF, and serum was determined by a 2-step fluorometric assay by Shire (Lexington, MA) using the 4-MUF substrate (MU-αldu-2S; Moscerdam Substrates, Oegstgeest, The Netherlands; order code M2) based on an assay described by Voznyi et al [12]. Briefly, tissue extracts were diluted in 0.2% bovine serum albumin (BSA) (pH and heat treated to inactivate lysosomal enzymes, supplemented with 0.004% sodium azide) and the substrate was desulfated by duplicate incubations of 10 μL of diluted sample with 20 μL of 1.25 mM substrate for 4 hours at 37°C. After I2S inhibition by the addition of 20 μL phosphate/citrate buffer (0.2M Na_2_HPO_4_/0.1 M citric acid, 0.02%s sodium azide, pH 4.7), samples were incubated with 10 μL of lysosomal enzymes purified from bovine testis for 24 hours at 37°C. This second reaction liberated 4-MUF from the substrate previously desulfated in the first reaction. The second reaction was stopped by the addition of 200 μL of stop buffer (0.5M NaHCO_3_/ Na_2_CO_3_, pH 10.7 with 0.025% Triton X-100) to each sample. Fluorescence was measured in 96-well fluorometry plates using SpectroMax M2 fluorescent plate reader (Molecular Devices Corporation, Sunnyvale, CA), and results were calculated using 4-methylumbelliferone (MP Biomedicals, Santa Ana, CA, part# 152475) as a standard with a range of 11.719–1500 pmol/L.

#### Protein Concentration Analysis by Enzyme Linked Immunosorbent Assay (ELISA)

I2S concentration in tissue extracts was determined by an I2S-specific ELISA by Shire (Lexington, Massachusetts). 96-well plates (Maxisorp Immuno-plate; Sigma-Aldrich, St Louis, MS, Nalge Nunc part 442404) were coated with 10 μg/mL of purified goat anti-I2S antibody (B347) and exposed to dilutions of tissue extract samples. After incubation for 1 hour, a polyclonal rabbit anti-I2S antibody (TK109) was added to the wells at a concentration of 0.67 μg/mL. After an additional hour of incubation, a goat anti-rabbit IgG conjugated with horseradish peroxidase (Promega, Madison, WI part# W4011) was added for a 30-minute incubation. Tetramethylbenzidine substrate (TMB Peroxidase EIA Substrate Kit; BioRad, Philadelphia, PA part# 172–1067) was then added to develop the plate, and absorbance at 450 nm was detected using a reference wavelength of 655 nm. Samples were compared to a standard curve using idursulfase (Shire, Lexington, MA, lot FDB04-003) with a working range of 0.156–20 ng/mL.

#### Normalization by bicinchoninic acid (BCA) Method

Results for ELISA and activity were normalized to total protein in tissue extracts as determined by BCA assay (BCA Assay; Pierce Biotechnology, Rockford, IL part #23227). I2S activity results for each sample were expressed as nM/h/mg protein or nU/mg protein, while ELISA results were expressed as ng/mg protein.

#### Pharmacokinetic Assessments

Serum and CSF concentration-time data from individual animals were analyzed using a non-compartmental analysis method as implemented in the Win-Nonlin 5.2 program (Pharsight Corp., Mountain View, CA). The following pharmacokinetic parameters were observed or calculated: maximum observed serum concentration (*C*_*max*_); time of *C*_*max*_ (*T*_*max*_); area under the concentration-time curve from time 0 to the last sampling with a concentration >lower limit of quantitation (*AUC*_*last*_) as calculated by the linear up/logarithmic down trapezoidal summation method; area under the serum concentration-time curve extrapolated to infinity (*AUC*_*inf*_) calculated by the linear up/logarithmic down trapezoidal summation method; mean residual time derived from time 0 to infinity (*MRT*_*inf*_); terminal half-life (*t½*) calculated as 0.693/λZ (where λZ is the apparent terminal rate constant derived from the slope of the log-linear regression of the log-linear terminal portion of the plasma concentration-time curve).

## Results

The endogenous or pre-dose levels (time 0) of I2S in the CSF and serum, measured prior to idursulfase-IT dosing in all animals, and the endogenous I2S levels in the brain tissues, spinal cord, liver and kidneys, measured from the 2 untreated controls are shown in Table 1. Pre-dose I2S activity in the serum was about 9-fold higher than that in the CSF (748 vs 85.4 nU/mL). Endogenous I2S concentrations in the brain structures ranged from 5.3–15.8 ng/mg protein. The lowest I2S concentration was seen in the cerebellum (5.3 ng/mg protein), and the highest in the spinal cord (15.8 ng/mg protein).

**Table 1:** Basal levels of I2S in selected tissues.

Tissue/Sample	Mean (±SD)	n	CV%	Assay
CSF, nU/mL	88.4 ±29.0	12	33.0	4-MUF
Spinal cord, ng/mg protein	15.8 ±5.4	9	33.9	ELISA
Cerebral cortex, ng/mg protein	9.1 ±1.8	12	19.9	ELISA
White matter, ng/mg protein	6.9 ±3.3	16	47.8	ELISA
Serum, nU/mL	748 ±334	12	45.0	4-MUF
Liver, ng/mg protein	1.2 ±0.16	6	13.3	ELISA
Kidney, ng/mg protein	4.6 ±0.85	2	18.5	ELISA

4-MUF, 4-methylumbelliferyl-α-iduronate-2-sulfate; CV, coefficient of variation; ELISA, enzyme linked immunosorbent assay; I2S, iduronate-2-sulfatase; SD, standard deviation.

The validated 4-MUF assay cannot differentiate between endogenous and exogenous I2S, and substantial levels of endogenous I2S activity were detected in the serum (847 nU/mL) and CSF (85.4 nU/mL) prior to idursulfase-IT administration. These values were respectively subtracted from the bioactivity measured in the serum and CSF samples post IT-L dosing. Protein concentrations (ng/mL) were not treated in the same manner, because the human idursulfase-IT-specific ELISA recognized the dosed I2S protein only. In fact, protein concentrations were not measurable in most of serum and CSF samples collected either prior to dose or in the control animals. Linear regressions of protein concentrations (ng/mL) versus enzyme activities (nU/mL) indicated positive correlations, *y* = 2.14*x* + 688 with an *r*^2^ = 0.865 (*P*<0.0001) for serum, and *y* = 0.453*x* + 135,000 *r*^2^ = 0.676 (*P* <0.0001) for CSF.

### Uptake of I2S from CSF to Spinal Cord

**Fig 3:**
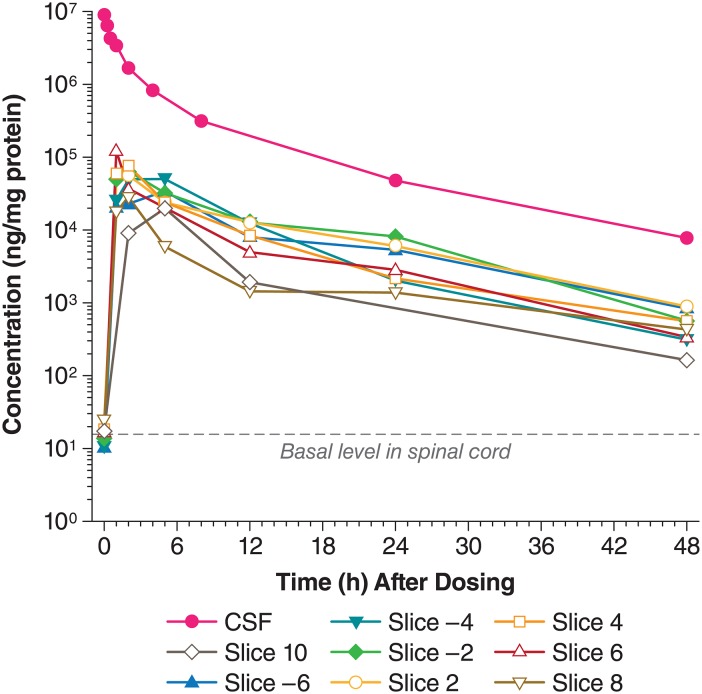
I2S concentrations in different levels of the spinal cord and in the CSF after IT-L administration. Note: An outlier in Slice 2 was 23-fold higher than the mean from all other slices and was not included in the mean calculation. The concentration-time data for the CSF is from Xie et al [10]. CM, cisterna magna; CSF, cerebrospinal fluid; I2S, iduronate-2-sulfatase; IT-L, intrathecal-lumbar.

I2S appeared in the spinal cord immediately after IT-L administration, with peak concentrations reached in 1–5 hours for most of the spinal sections (Fig 3). The values of maximum observed concentration (*C*_*max*_) were 60,533; 82,160; 58,165; 51,559; 95,741; 33,940 and 12,336 ng/mg protein in sections +2, -2, +4, -4, +6, -6, and +8, respectively, which were about 1000 to 10,000 times greater than the endogenous levels. The highest concentration was seen around the tip of dosing catheter at sections 2 and -2 of the spinal cord. Concentration gradients around the catheter tip existed, especially 6–24 hours after dosing. I2S concentrations were approximately 100 times greater than the basal level 48 hours after administration. The half-life of I2S in the spinal cord ranged from 6.2–8.9 hours (S1 Table). The values of the area under the concentration-time curve (*AUC*) from 0–48 hours after 30 mg IT-L dosing for the different spinal cord sections were about 150 to 900-fold greater than the endogenous levels (112,288–685,047 ng/mg vs 758 ng/mg).

### Uptake of I2S from CSF to brain tissues

#### Cerebral cortex

**Fig 4:**
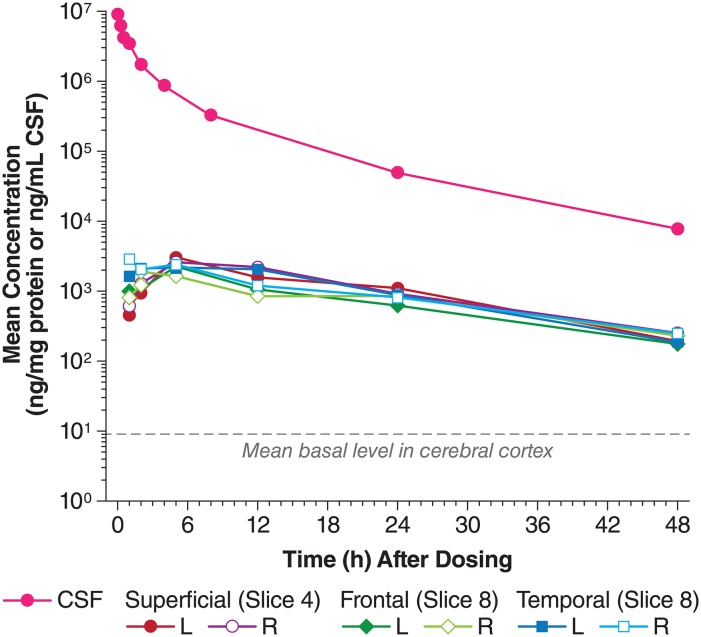
Comparison of I2S concentrations (ELISA) in the cerebral cortex and the CSF after IT-L administration. CM, cisterna magna; CSF, cerebrospinal fluid; I2S, iduronate-2-sulfatase; IT-L, intrathecal-lumbar.

I2S appeared in the cerebral cortex as early as 1 hour (the earliest sampling time point) after IT-L administration. Peak concentrations were found between 2 and 5 hours (Fig 4). At 1 hour post IT-L administration, I2S uptake was higher in the temporal cortex (1063 and 1542 ng/mg protein in the left and right sides from slice 8) than in the frontal cortex (540 and 546 ng/mg protein in the left and right sides from the same slice), and much higher than those in the left and right cortex (277 and 360 ng/mg protein from slice 4). However, the I2S concentrations in the cortex tissues were very similar (range 1285–2465 ng/mg protein (for punches taken from slices 8 and 4) at 5 hours post IT-L dosing, and the concentration-time curves overlapped each other. I2S cortex concentrations were 30- to 50-fold, and 6- to 8-fold greater than the endogenous levels at 24 and 48 hours after IT-L administration.

The terminal elimination phase in all these cortex tissues obeys typical first-order kinetics. Because I2S elimination from these cortex tissues was similar, non-compartmental parameters of individual cortex tissue were pooled and summarized in Table 2. The values of *C*_*max*_, *AUC*_*last*_, and *AUC*_*inf*_ were expected to be different depending on whether the I2S concentrations were expressed as protein amount or as enzyme activity. However, the secondary non-compartmental pharmacokinetic parameters analyzed by I2S concentration-time profiles were very similar whether measured by ELISA or enzyme activity (4-MUF).

**Table 2:** Comparison of non-compartmental pharmacokinetic parameters for the cerebral cortex measured by ELISA and 4-MUF (n = 2).

	ELISA		Enzyme Activity (4-MUF)	
	Units	Mean (±SD)	Units	Mean (±SD)
***t***_***1/2***_	h	13.1 ±2.4	h	10.5 ±1.0
***T***_***max***_	h	3.8 ±1.8	h	3.3 ±1.9
***C***_***max***_	ng/mg protein	2.5 ±0.4	nU/mg protein	1615 ±442
***AUC***_***last***_	h*ng/mg protein	48.5 ±8.2	h*nU/mg protein	25,654 ±4077
***AUC***_***inf***_	h*ng/mg protein	52.7 ±7.7	h*nU/mg protein	26,707 ±3982
***MRT***_***inf***_	h	19.4 ±2.3	h	15.5 ±0.7

4-MUF, 4-methylumbelliferyl-α-iduronate-2-sulfate; *AUC*_*inf*_, area under the concentration-time curve extrapolated to infinity; *AUC*_*last*_, area under the concentration-time curve from time 0 to the last sampling with a concentration >lower limit of quantitation; *C*_*max*_, maximum observed concentration; ELISA, enzyme linked immunosorbent assay; *MRT*_*inf*_, mean residual time in hours derived from time 0 to infinity; SD, standard deviation; *t½*, terminal half-life; *T*_*max*_, time of occurrence of *C*_*max*_.

#### White matter

**Fig 5:**
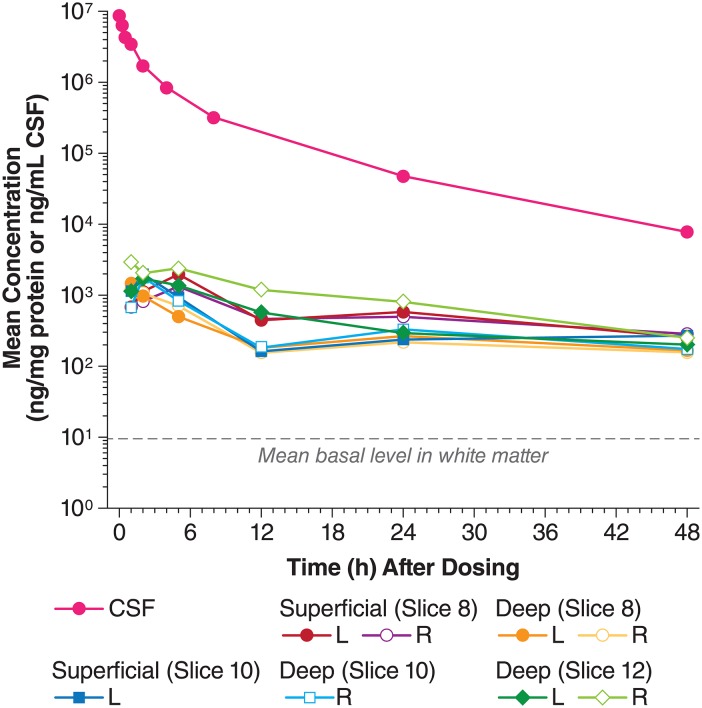
Comparison of I2S concentrations in the white matter and the CSF after IT-L administration. CM, cisterna magna; CSF, cerebrospinal fluid; ELISA, enzyme linked immunosorbent assay; I2S, iduronate-2-sulfatase; IT-L, intrathecal-lumbar.

I2S appeared in the white matter as early as 1 hour after IT-L administration, and peak concentrations were observed around 2 and 5 hours. I2S uptake was similar in different locations and depths in the brain (Fig 5). I2S concentrations were approximately 50-, 10-, 13-, and 7-fold, higher than the endogenous idursulfase levels at 5, 12, 24, and 48 hours after IT-L dosing, respectively. The terminal elimination phase followed first-order kinetics. As with the cortex, non-compartmental parameters of individual tissues were pooled together, as the elimination of I2S from white matter tissues was similar (Table 3). The secondary pharmacokinetic parameters from ELISA and 4-MUF data were very similar. The *T*_*max*_ for white matter was shorter compared to that seen in the cortex (2.1 vs 3.8 hours), and *t*_*1/2*_ was longer in the white matter than in the cortex (23.3 vs 13.1 hours). The I2S *C*_*max*_ was lower in the white matter than in the cortex (1.5 vs 2.5 h*ng/mg protein), as was the *AUC*_*inf*_ (29.0 vs 52.7 h*ng/mg protein).

**Table 3:** Comparison of non-compartmental pharmacokinetic parameters for the white matter measured by ELISA and 4-MUF (n = 2).

	ELISA		Bioactivity (4-MUF)	
	Units	Mean (±SD)	Units	Mean (±SD)
***t***_***1/2***_	h	23.3 ±9.1	h	22.3 ±2.7
***T***_***max***_	h	2.1 ±1.2	h	1.6 ±0.5
***C***_***max***_	ng/mg protein	1.5 ±0.4	nU/mg protein	887 ±334
***AUC***_***last***_	h*ng/mg protein	21.2 ±6.1	h*nU/mg protein	9011 ±2813
***AUC***_***inf***_	h*ng/mg protein	29.0 ±9.1	h*nU/mg protein	11,404 ±3773
***MRT***_***inf***_	h	34.6 ±10.3	h	27.5 ±5.4

4-MUF, 4-methylumbelliferyl-α-iduronate-2-sulfate; *AUC*_*inf*_, area under the concentration-time curve extrapolated to infinity; *AUC*_*last*_, area under the concentration-time curve from time 0 to the last sampling with a concentration >lower limit of quantitation; *C*_*max*_, maximum observed concentration; ELISA, enzyme linked immunosorbent assay; *MRT*_*inf*_, mean residual time derived from time 0 to infinity; SD, standard deviation; *t½*, terminal half-life; *T*_*max*_, time of occurrence of *C*_*max*_.

#### Consistency of I2S protein concentrations with bioactivity in all brain structures

**Fig 6:**
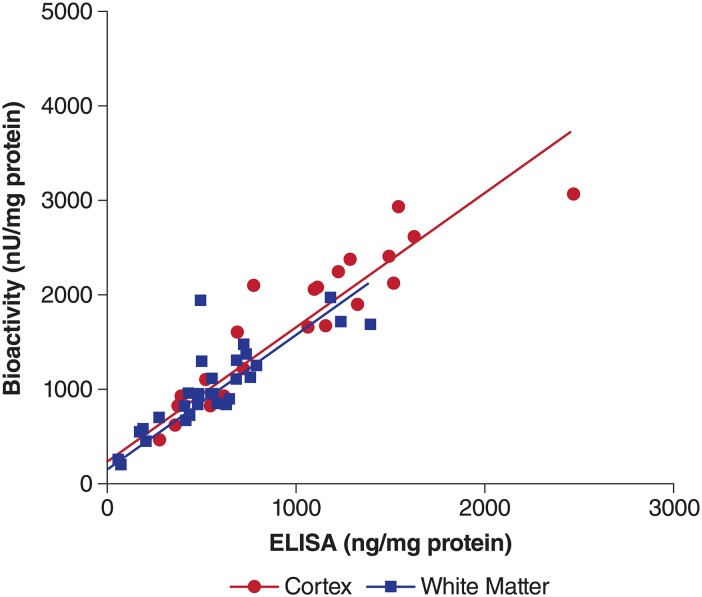
Correlation and linear regression of I2S concentrations measured by ELISA and bioactivity (4-MUF) assays. 4-MUF, 4-methylumbelliferyl-α-iduronate-2-sulfate; ELISA, enzyme linked immunosorbent assay; I2S, iduronate-2-sulfatase.

The correlation of 2 sets of concentration results, and linear regressions on the ELISA concentrations and 4-MUF bioactivities are shown in Fig 6. The lines from the regressions on each set of data for cortex and white matter, overlapped each other. The coefficient of determination (*r*^*2*^*)* values for cortex and white matter were 0.909 (*P*<0.0001) and 0.838 (*P*<0.0001), respectively.

### Uptake of I2S from CSF to Peripheral Organs

**Fig 7:**
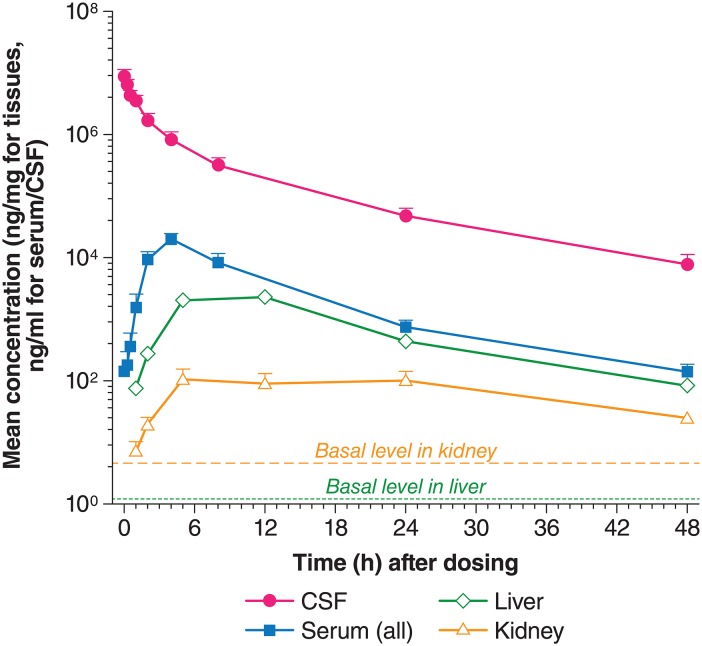
Comparison of I2S concentrations in the kidneys, liver, CSF, and serum after IT-L administration. ELISA, enzyme linked immunosorbent assay; IT-L, intrathecal-lumbar.

I2S appeared in the serum 5 min after IT-L administration. The serum concentrations increased quickly, and reached peak values about 3–4 hours following IT-L administration. Serum concentrations were continuously detectable for 48 hours post IT-L dosing. Serum bioavailability after I2S dosing has been estimated about 80% at this dose level [10]. I2S appeared in the liver and kidney 1 hour post dose (the earliest time point for sampling). The peak I2S concentration was around 12 hours in the liver, and 5 hours in the kidneys. I2S was continuously higher than basal idursulfase levels in these organs for 48 hours (the last time point for sampling) after IT-L administration (Fig 7). Liver exposure to I2S was >10-fold higher than that in the kidneys, with *C*_*max*_ values 2253 and 109 ng/mg protein, respectively. The value of *AUC*_*inf*_ in the liver was about 10-fold larger than in the kidneys (41,701 vs 4028 h*ng/mg protein) (Table 4).

**Table 4:** Non-compartmental pharmacokinetic parameters for the liver and kidneys measured by ELISA (n = 2).

	Kidney	Liver
*t*_*1/2*_, h	11.9	8.6
*T*_*max*_, h	5	12
*C*_*max*_, ng/mg protein	109	2253
*AUC*_*last*_, h*ng/mg protein	3597	40,682
*AUC*_*inf*_, h*ng/mg protein	4028	41,701

*AUC*_*inf*_, area under the concentration-time curve extrapolated to infinity; *AUC*_*last*_, area under the concentration-time curve from time 0 to the last sampling with a concentration >lower limit of quantitation; *C*_*max*_, maximum observed concentration; ELISA, enzyme linked immunosorbent assay; *t½*, terminal half-life in hours; *T*_*max*_, time in hours of occurrence of *C*_*max*_.

## Discussion and Conclusions

Although idursulfase has been approved for enzyme replacement therapy of Hunter syndrome, intravenously administered idursulfase does not treat the neurological manifestations, due to its low CNS bioavailability [5,7]. Overcoming the blood brain barrier presents many difficulties and is the subject of many drug delivery techniques currently in development [13, 14]. Injection of protein therapeutics directly into the CSF bypasses the blood-brain barrier altogether and has been successfully used in cancer chemotherapy and pain management and also for the delivery of recombinant enzymes to animal models of lysosomal diseases [15].

Felice et al [8] have shown that monthly idursulfase-IT administered intrathecally (in addition to weekly intravenous idursulfase) is generally well tolerated in cynomolgus monkeys. They showed, using immunohistochemistry with anti-human idursulfase monoclonal antibodies, that 30-mg and 100-mg doses of idursulfase-IT localized I2S to large numbers of cerebral neurons, meningeal and glial cells in surface neurons next to the meninges, and in deep neurons adjacent to white matter, as well as to meningeal and glial cells, and neurons in the spinal cord. This was in contrast to the control animals (no treatment) and those animals following a 4-week, no treatment recovery period, which showed no localization [8]. Idursulfase activity (measured by the 4-MUF method) showed I2S was above baseline in the brain and spinal cord in animals in the 30- and 100-mg dose groups [8]. Calias et al [9], using ^124^I-labelled idursulfase and positron emission tomography, observed the presence of labeled I2S in the brain parenchyma and the spinal cord of cynomolgus monkeys after IT-L administration. Using a monoclonal antibody specific to human I2S, widespread cellular deposition of I2S in the neurons of the cerebrum, cerebellum, brainstem, and spinal cord in a dose dependent manner was demonstrated. I2S was observed to be present in the surface gray matter and in neurons of the thalamus, hippocampus, caudate nucleus, and spinal cord. IT-L administration was also shown to deliver I2S successfully to the spinal cord, whereas intraventricular administration only resulted in low I2S deposition to the spinal cord.

Xie et al [16], investigated the pharmacokinetics and bioavailability of IT-L administered idursulfase-IT in cynomolgus monkeys, and confirmed that IV-administered idursulfase provided minimal I2S to the CNS, compared with about 8000–30,000 times greater CSF I2S *C*_*max*_ concentrations observed after IT-L administration (10 mg and 30 mg doses). The long residence time of the administered I2S in the CNS (mean *t*_*1/2*_ = 10 hours for 30-mg dose) suggests that the enzyme would be effective at eliminating accumulated GAG from the CNS when translated to a clinical, human environment for patients with Hunter syndrome.

In this study, and following on from Xie et al’s work, I2S levels were measured in the CSF, spinal cord, a variety of brain tissues, serum, and peripheral organs of cynomolgus monkeys following IT-L administration. In addition, idursulfase enzyme activity was analyzed using the 4-MUF substrate degradation method. Tissue ELISA confirmed uptake to the brain, spinal cord, kidneys, and liver in a time-dependent manner after IT-L dosing of I2S. The enzyme activity assay confirmed that the I2S within the tissues was bioactive.

The various brain tissues and sections of the spinal cord had differing levels of endogenous I2S and these tissues also varied in the degree of uptake of exogenous I2S. Predose idursulfase activity in the serum of the monkeys was about 9-fold higher than that in the CSF (748 versus 85.4 nU/mL). Endogenous idursulfase protein concentrations in the brain structures were in a range from 5.3–15.8 ng/mg protein. The lowest concentration was seen in the cerebellum (5.3 ng/mg protein), and the highest in the spinal cord (15.8 ng/mg protein). Idursulfase concentrations in the liver and kidneys were 1.2 and 4.6 ng/mg protein, respectively.

I2S appeared in the spinal cord in a time-dependent manner immediately after IT-L dosing, with peak concentrations (*C*_*max*_) of 1000–10,000-fold greater than the basal levels being achieved around 2 and 5 hours after dosing, and levels were still approximately 100-fold greater than the basal level after 48 hours. I2S *t*_*1/2*_ in the spinal cord was about 7 hours, and the *AUC* from 0–48 hours were about 150- to 900-fold greater than the endogenous levels.

The appearance of I2S in the cerebral cortex and white matter was also time-dependent, detected as early as 1 hour post dosing, with *T*_*max*_ around 2 and 5 hours. Twenty-four hours after IT-L administration, I2S levels remained 30–50-fold above endogenous levels in the cerebral cortex, and 13-fold greater in the white matter. White matter I2S *T*_*max*_ was shorter compared with cerebral cortex (2.1 vs 3.8 hours), although I2S *t*_*1/2*_ was longer (23.3 vs 13.1 hours). White matter showed lower exposure to I2S compared with the cerebral cortex (*AUC*_*inf*_ 29.0 vs 52.7 h*ng/mg protein). As the enzyme was administered into the subarachnoid cavity of the spinal cord, the levels of exogenous I2S were highest around the catheter, after which the enzyme diffused outwards through the CSF to the brain. The circulation of the CSF presumably carries the I2S to the cerebral cortex rapidly with peak concentrations reached in 2–5 hours. Protein delivery to the surface of the brain and then on to the deeper parts is thought to depend on active transport systems and involves interactions with neuronal mannose 6-phosphatase receptors located on the neuronal membrane [17], followed by endocytosis and axonal transport [9, 18, 19]. It is therefore not unexpected that exogenous I2S will in occur in greater amounts in the superficial areas of the cortex, and that penetration of I2S into deeper areas of the brain (white matter) might result in lower exposure over the 48 hours following administration. I2S was detected rapidly in the white matter, but the amount and activity of the I2S was lower than that seen in the cerebral cortex.

The linear regressions on the concentration data measured from the same samples using ELISA and 4-MUF bioactivity for cortex and white matter, overlapped each other, indicating a good correlation between the protein amount and bioactivity, as well as a similar uptake and elimination mechanism in these structures.

This study lends further evidence in support of intrathecal administration of idursulfase-IT as a potential treatment for the cognitive manifestations of Hunter syndrome, demonstrating that intrathecal administration bypasses the blood brain barrier allowing the recombinant enzyme to directly penetrate the CNS. Clinical trials using intrathecal delivery of idursulfase-IT are continuing in patients with Hunter syndrome to investigate this further (NCT00920647 [11] and NCT02055118).

An interesting feature of IT-L administration in cynomolgus monkeys is that there is passage of I2S from the CSF to the serum and thence to liver and kidneys, although the transition from CSF to liver is slow (T_*max*_ 12 hours). Xie et al reported (using the same animals as in this paper) that 30 mg idursulfase by IT-L administration lead to a serum *C*_*max*_ approximately twice that of 0.5 mg/kg idursulfase administered intravenously [10]. The *C*_*max*_ value of I2S in the kidneys was 109 ng/mg protein after IT-L administration compared with basal levels of 4.6 ng/mg protein for endogenous I2S. For liver, the IT-L value was 2253 ng/mg protein versus a basal value of 1.2 ng/mg protein. Transposed to a human, clinical environment, this may be significant in terms of potentially helping to ameliorate the somatic symptoms of patients with Hunter syndrome undergoing intrathecal idursulfase enzyme replacement therapy [11]. Currently, somatic symptoms of Hunter syndrome are treated by intravenous idursulfase treatment [6, 20].

In conclusion, this study confirms that idursulfase-IT, when administered intrathecally at the lumbar level enters the cerebral cortex, white matter, spinal cord, and retains activity within the cells as determined by enzyme activity measurements and that intrathecal-lumbar treatment with recombinant iduronate-2-sulfatase may be considered for further investigation as a treatment for Hunter syndrome patients with neurocognitive impairment.

## Supporting Information

S1 Table Non-compartmental pharmacokinetic parameters for each section of the spinal cord (n = 2). Pharmacokinetic analysis was done only for the sections of spinal cord with the concentration values available for all 7 time points. *AUC*_*inf*_, area under the concentration-time curve extrapolated to infinity; *AUC*_*last*_, area under the concentration-time curve from time 0 to the last sampling with a concentration >lower limit of quantitation; *C*_*max*_, maximum observed concentration; I2S, *MRT*_*inf*_, mean residual time derived from time 0 to infinity; *t½*, terminal half-life; *T*_*max*_, time of occurrence of *C*_*max*_.
(DOCX)
