# Supplementary material for: Biodistribution of Idursulfase Formulated for Intrathecal Use (Idursulfase-IT) in Cynomolgus Monkeys after Intrathecal Lumbar Administration
Source: PLoS One. 2016 Oct 20;11(10):e0164765. doi: 10.1371/journal.pone.0164765 (PMC5072681; doi:10.1371/journal.pone.0164765)
Supplement: S1 Table — Pharmacokinetic analysis was done only for the sections of spinal cord with the concentration values available for all 7 time points. AUCinf, area under the concentration-time curve extrapolated to infinity; AUClast, area under the concentration-time curve from time 0 to the last sampling with a concentration >lower limit of quantitation; Cmax, maximum observed concentration; I2S, MRTinf, mean residual time derived from time 0 to infinity; t½, terminal half-life; Tmax, time of occurrence of Cmax. (DOCX) [file pone.0164765.s001.docx]

**Supporting Information**

**Table S1. Non-compartmental pharmacokinetic parameters for each section of the spinal cord (n=2)**

| **Section** | **2** | **- 2** | **4** | **- 4** | **6** | **- 6** | **8** |
| --- | --- | --- | --- | --- | --- | --- | --- |
| *t½*, h | 7.2 | 7.5 | 6.7 | 6.2 | 7.3 | 6.7 | 8.9 |
| *T_max_*, h | 2 | 2 | 1 | 2 | 1 | 5 | 1 |
| *C_max_*, ng/mg protein | 60,533 | 82,160 | 58,165 | 51,559 | 95,741 | 33,940 | 12,336 |
| *AUC_last_,* h×ng/mg protein | 619,473 | 685,074 | 400,367 | 567,270 | 322,894 | 486,392 | 112,288 |
| *AUC_inf_,* h×ng/mg protein | 729,336 | 693,491 | 403,950 | 570,759 | 326,978 | 489,245 | 115,289 |
| *MRT_inf_*, h | 23.4 | 10.3 | 7.5 | 7.7 | 7.7 | 10.7 | 11.8 |

Pharmacokinetic analysis was done only for the sections of spinal cord with the concentration values available for all 7 time points.

*AUC_inf_*, area under the concentration-time curve extrapolated to infinity; *AUC_last_*, area under the concentration-time curve from time 0 to the last sampling with a concentration >lower limit of quantitation; *C_max_*, maximum observed concentration; I2S, *MRT_inf_*, mean residual time derived from time 0 to infinity; *t½,* terminal half-life; *T_max_*, time of occurrence of *C_max_*.
